# Melanoma with in-frame deletion of *MAP2K1*: a distinct molecular subtype of cutaneous melanoma mutually exclusive from *BRAF*, *NRAS*, and *NF1* mutations

**DOI:** 10.1038/s41379-020-0581-5

**Published:** 2020-06-01

**Authors:** Erik A. Williams, Meagan Montesion, Nikunj Shah, Radwa Sharaf, Dean C. Pavlick, Ethan S. Sokol, Brian Alexander, Jeff Venstrom, Julia A. Elvin, Jeffrey S. Ross, Kevin Jon Williams, Julie Y. Tse, Mark C. Mochel

**Affiliations:** 1 Foundation Medicine, Inc., 150 Second Street, Cambridge, MA 02141 USA; 2 Department of Pathology, State University of New York Upstate Medical University, 766 Irving Avenue, Syracuse, NY 13210 USA; 3 Department of Physiology, Department of Medicine, Lewis Katz School of Medicine at Temple University, Philadelphia, PA 19140 USA; 4 Department of Pathology & Laboratory Medicine, Tufts University School of Medicine, 145 Harrison Ave, Boston, MA 02111 USA; 5 Departments of Pathology and Dermatology, Virginia Commonwealth University School of Medicine, Richmond, VA USA

**Keywords:** Melanoma, Cancer genomics

## Abstract

While the genomics of *BRAF*, *NRAS*, and other key genes influencing MAP kinase (MAPK) activity have been thoroughly characterized in melanoma, mutations in *MAP2K1 (MEK1)* have received significantly less attention and have consisted almost entirely of missense mutations considered secondary oncogenic drivers of melanoma. Here, we investigated melanomas with in-frame deletions of *MAP2K1*, alterations characterized as MAPK-activating in recent experimental models. Our case archive of clinical melanoma samples with comprehensive genomic profiling by a hybrid capture-based DNA sequencing platform was searched for *MAP2K1* genetic alterations. Clinical data, pathology reports, and histopathology were reviewed for each case. From a cohort of 7119 advanced melanomas, 37 unique cases (0.5%) featured small in-frame deletions in *MAP2K1*. These included E102_I103del (*n* = 11 cases), P105_A106del (*n* = 8), Q58_E62del (*n* = 6), I103_K104del (*n* = 5), I99_K104del (*n* = 3), L98_I103del (*n* = 3), and E41_F53del (*n* = 1). All 37 were wild type for *BRAF*, *NRAS*, and *NF1* genomic alterations (“triple wild-type”), representing 2.0% of triple wild-type melanomas overall (37/1882). Median age was 66 years and 49% were male. The majority arose from primary cutaneous sites (35/37; 95%) and demonstrated a UV signature when available (21/25; 84%). Tumor mutational burden was typical for cutaneous melanoma (median = 9.6 mut/Mb, range 0–35.7), and frequently mutated genes included *TERTp* (63%), *CDKN2A* (46%), *TP53* (11%), *PTEN* (8%), *APC* (8%), and *CTNNB1* (5%). Histopathology revealed a spectrum of appearances typical of melanoma. For comparison, we evaluated 221 cases with pathogenic missense single nucleotide variants in *MAP2K1*. The vast majority of melanomas with missense SNVs in *MAP2K1* showed co-mutations in *BRAF* (58%), *NF1* (23%), or *NRAS* (18%). In-frame deletions in *MAP2K1*, previously shown in experimental models to be strongly MAPK-activating, characterized a significant subset of triple wild-type melanoma (2.0%), suggesting a primary oncogenic role for these mutations. Comprehensive genomic profiling of melanomas enables detection of this alteration, which may have implications for potential therapeutic options.

## Introduction

Most melanomas harbor driver mutations which promote activation of the MAP Kinase pathway, most frequently through mutations in *BRAF*, *NRAS*, or *NF1* [1–4]. Increasingly, gene fusions involving various kinases, including *ALK*, *ROS1*, *BRAF*, *RAF1*, *NTRK1*, and *NTRK3* have been identified within melanomas, and these fusion-positive melanomas often display spitzoid morphology [5–9]. In addition to these primary driver mutations, various secondary oncogenic driver mutations have been identified, including gain of function alterations, such as *TERT* promoter mutation and *CDK4* amplification, and loss of function alterations, such as *CDKN2A*, *TP53*, and *PTEN* mutations [2].

Melanomas also contain *MAP2K1* (*MEK1*) mutations, with prior reports consisting almost entirely of missense mutations characterized as secondary oncogenic driver mutations [2, 10, 11]. Most reported *MAP2K1* mutations have accompanied other driver mutations of melanoma including *BRAF*, *NRAS*, and *NF1* [4, 10–12]. Prompted by recent studies of mutual exclusivity among *BRAF* mutation and *MAP2K1* mutation in Langerhans cell histiocytosis (LCH) [13–15], as well as work showing distinct functional classes of *MAP2K1* mutations [16], we probed a database of clinical specimens with comprehensive genomic profiles for the relationship between *MAP2K1* mutations and other driver mutations in melanoma. Herein, we report a series of melanomas with *MAP2K1* in-frame deletions distinct from other driver mutations of melanoma.

## Materials and methods

### Cohort and genomic analyses

Cases evaluated for this study had undergone comprehensive genomic profiling (CGP) performed in a Clinical Laboratory Improvement Amendments-certified, College of American Pathologists-accredited laboratory (Foundation Medicine, Inc., Cambridge, MA, USA). The Western Institutional Review Board granted approval for this study (Protocol No. 20152817), including a waiver of informed consent and a HIPAA waiver of authorization. Briefly, hematoxylin and eosin (H&E)-stained histologic slides were reviewed to verify the presence of diagnostic lesional tissue. From formalin-fixed, paraffin-embedded tissue blocks, ≥60 ng of DNA was extracted from 40 μm sections. The samples were assayed by CGP using adapter ligation, and hybrid capture was performed for exons from 287 (version 1) to 315 (version 2) cancer-related genes plus select introns from 19 (version 1) to 28 (version 2) genes frequently rearranged in cancer (Supplemental Table 1). Sequences were analyzed for all classes of genomic alterations including short variant alterations (base substitutions, insertions, and deletions), copy number alterations (focal amplifications and homozygous deletions), and select gene fusions or rearrangements, by methods previously described [17–19]. A somatic-germline-zygosity computational method was applied for somatic, germline, and artifact determination; the method fit an optimal copy number model to the log-ratio and minor allele frequency data and compared the observed variant allele frequency against that of the model’s expected variant allele frequency [18]. Tumor mutational burden (TMB, mutations/Mb) was determined on 0.8–1.1 Mbp of sequenced DNA [19]. Microsatellite instability (MSI) was determined on up to 114 loci [20].

Cell-free circulating tumor DNA (ctDNA) was evaluated on 315 blood specimens (“liquid biopsy”) collected from 307 patients with clinical history of melanoma using the hybrid capture-based Illumina Hi-Seq technology. Maximum somatic allele frequency was used to estimate the fraction of ctDNA, per methods previously described [21, 22].

### Mutational signatures

For all samples containing at least 20 nondriver somatic missense alterations, mutational signatures were assessed through analysis of the trinucleotide context and profiling by Sanger COSMIC signatures of cancer mutational processes [23]. A positive signature was designated for samples with at least a 40% fit to a mutational process [23]. The COSMIC UV signature is characterized by C>T and CC>TT base substitutions at dypirimidine sites [24].

### Clinical-pathological analysis of melanoma cohort harboring in-frame deletions in MAP2K1

From a total of 7119 consecutive routine clinical melanoma specimens, each from a different patient, the cohort of melanomas harboring in-frame deletions in *MAP2K1* comprised 37 cases. Samples which underwent assays with CGP (Foundation Medicine, Cambridge, MA, USA) were collected from patients receiving clinical care at other institutions. Clinicopathological data including patient age, gender, tumor site, tumor diameter, and stage were extracted from accompanying pathology reports.

H&E stained sections from each of the 37 cases were assessed retrospectively by two board-certified dermatopathologists (JYT, MCM) and an additional board-certified anatomic pathologist (EAW). Histologic parameters assessed on primary tumors included configuration at low magnification, symmetry, presence and patterns of epidermal involvement, ulceration, pattern of dermal growth, Breslow depth, mitotic rate, grade of solar elastosis [25], and tumor infiltrating lymphocytes. Primary and metastatic cases were assessed for cytologic features, including predominant cytomorphology (epithelioid vs. spindled), cytoplasmic color and abundance, chromatin quality, nucleolar prominence, and degree of pleomorphism. Accompanying pathology reports were utilized for diagnostically corroborating details, including immunohistochemical findings and melanoma history.

Quantitative data were analyzed using the Fisher exact test owing to the categorical quality of the data and the size of the cohort. For TMB comparison between two groups, the nonparametric Mann–Whitney *U* test was used. A two‐tailed *P* value of <0.05 was considered to be statistically significant; the Bonferroni correction was applied for multiple simultaneous comparisons.

## Results

### Clinical-pathologic features

Of 7119 melanomas with prior hybrid capture-based DNA sequencing, 37 distinct cases (0.5%) featured small in-frame deletions that resulted in known or likely activation of *MAP2K1*. Among patients with *MAP2K1* in-frame deleted melanomas, the ages ranged from 28 to 89 years, with a median of 66 years. There were 18 males and 19 females. Age and gender did not show significant differences compared with the melanoma cohort overall. Nearly all cases were clinically advanced: most cases were documented at stage IV (*n* = 25 of 37; 68%), with the majority of the remaining cases documented at stage IIIA-C (*n* = 10 of 37; 27%). The other two cases were stage IIA and IIC.

Sequencing was performed on four primary cutaneous melanomas (Table 1), three primary site recurrences, and 30 metastatic disease samples. Of the metastatic samples, sites included regional lymph nodes (*n* = 5), in-transit metastasis (*n* = 1), and distant lymph nodes (*n* = 6). Additional distant metastatic sites included skin (subcutaneous [n = 6], dermal [*n* = 2]), lung (*n* = 5), brain (*n* = 2), and one each involving liver, omentum, and pericardium. Thirty-five cases were consistent with either primary cutaneous melanoma or metastatic melanoma from a cutaneous primary, while two cases were of unknown primary site.

**Table 1:** Clinical and pathologic features of primary cutaneous *MAP2K1* in-frame deletion melanomas.

Case	Gender	Age (years)	Location	Diameter (mm)	Subtype	Depth (mm)	Mitoses/mm^2^	Cytology
1	M	41	Back	12	Nodular	3.3	2	Epithelioid and spindled
2	M	28	Back	15	Superficial spreading	4.0	2	Epithelioid
3	F	89	Leg	20	Superficial spreading	5.0	13	Epithelioid
4	M	60	Leg	4	Nodular	1.3	1	Epithelioid

**Fig. 1:**
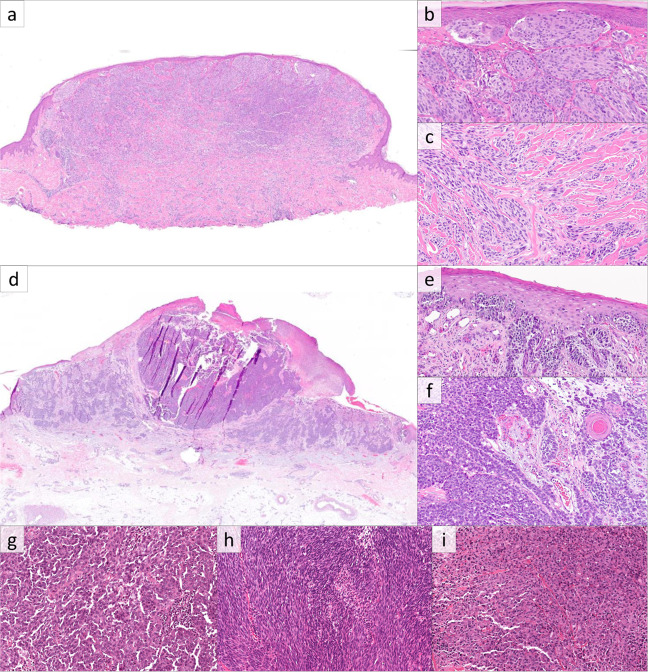
Melanoma with *MAP2K1* in-frame deletion. **a** Histopathologic examination of case 1 reveals a polypoid proliferation of melanocytes with a slight epidermal collarette (H&E, ×20). **b**, **c** Case 1 shows expansile nested growth along the epidermis with associated epidermal effacement, and elongated nests in the deep aspect. **d** Case 3 is a melanocytic tumor with plaque-like and tumorigenic components (H&E, ×200). **e** The radial growth phase of Case 3 shows a confluent proliferation of atypical melanocytes along the basal epidermis (H&E, ×200). **f** The deep aspect of the tumorigenic component consists of nodules of atypical epithelioid cells (H&E, ×200). Metastatic lesions showed predominantly epithelioid cytomorphology (**g**), with occasional cases displaying spindled (**h**), and rhabdoid morphology (**i**). (H&E, ×400).

Histopathologic evaluation of melanomas revealed variable morphologies. Of the four primary cutaneous melanomas (excluding the primary site recurrences), two were superficial spreading type, and two were nodular type (Fig. 1). All primary tumors showed exophytic nodular components with irregular nested growth at the base and marked asymmetry (Fig. 1a, d). All showed epidermal involvement, with the two superficial spreading melanomas showing extension beyond the dermal component (Fig. 1b, e). Two lesions were ulcerated, three showed confluent growth along the basal epidermis with rete effacement, three showed pagetoid growth, and two had expansile intraepidermal nests (Fig. 1b). Dermal components consisted of variably sized nodules and nests in all cases (Fig. 1f), and one case contained elongated nests in some foci (Fig. 1c). Tumor infiltrating lymphocytes were present and non-brisk in all cases. As defined by the World Health Organization [25], solar elastosis scores were 0 in one case, 2 in two cases, and 3 in one case. Cytomorphologically, three were epithelioid and one was an admixture of epithelioid and spindled melanocytes. Melanocytes had variable amounts of cytoplasm which was amphophilic in three cases and densely eosinophilic in one case. Only one case showed cytoplasmic pigmentation, which was focal. Nuclear size was medium to large, chromatin was heterogenous (of varying density), nucleoli were variably prominent, and nuclear pleomorphism was moderate to severe.

Among the 33 recurrent and metastatic tumors, 28 cases (85%) showed epithelioid cytomorphology (Fig. 1g), while five (15%) had mixed epithelioid and spindled cells (Fig. 1h). Among the epithelioid cases, three had rhabdoid cytology. Of note, each case with rhabdoid cytology was *MAP2K1* Q58_E62del mutant (Fig. 1i). Cytoplasm tended to be moderate to abundant in amount, and only seven cases (21%) contained cytoplasmic melanin which was focal in five cases and diffuse in two. Nuclear size was medium to large, and 13 cases (39%) had prominent nucleoli, with the remaining cases showing small to indistinct nucleoli.

### Comprehensive genomic profiling

**Fig. 2:**
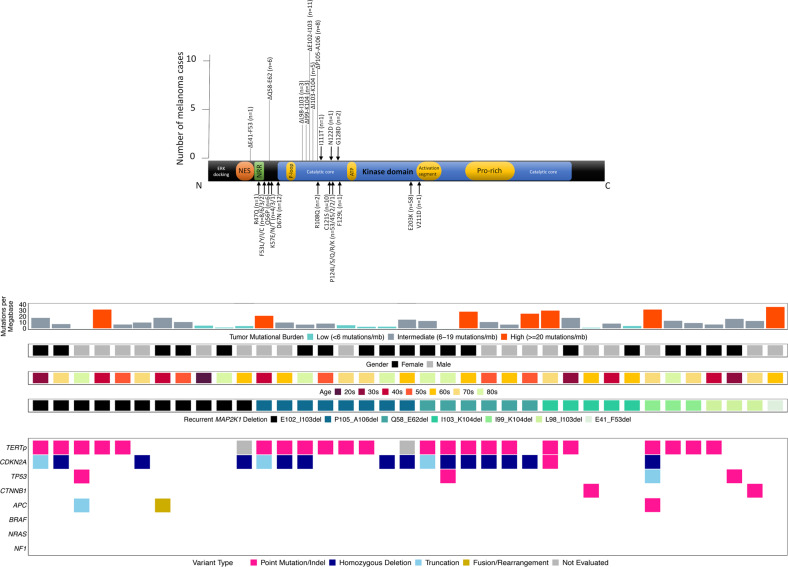
Molecular profiles of melanoma with *MAP2K1* in-frame deletion. **a** Schematic of functional domains of *MAP2K1* (transcript NM_002755), to include number of cases with each in-frame deletion (black bars; length~number of cases) and sites of pathogenic missense mutations (black arrows). The majority of in-frame deletions are present in the catalytic core. **b** Summary of clinical features and molecular alterations in *MAP2K1* in-frame deletion melanomas. NES nuclear export signal, NRR negative regulatory region, ATP ATP binding site, Pro rich proline-rich domain, *TERTp* *TERT* promoter.

*MAP2K1* in-frame deletions included E102_I103del (*n* = 11 cases), P105_A106del (*n* = 8), Q58_E62del (*n* = 6), I103_K104del (*n* = 5), I99_K104del (*n* = 3), L98_I103del (*n* = 3), and E41_F53del (*n* = 1) (Fig. 2a). Most deletions involved the portion of the gene corresponding to the kinase domain [16]. All 37 were wild type for *BRAF*, *NRAS*, and *NF1* genomic alterations (“triple wild-type”) (Fig. 2b), representing 2.0% of triple wild-type melanomas overall (37/1882) and showing significant enrichment for the triple wild-type subcategory compared with the melanoma cohort overall (*p* < 0.0001, Fisher’s exact test). In melanomas with in-frame deletions, median age was 66 years, 51% were female, and the large majority were confirmed by history to be cutaneous primary (35/37; 95%) and demonstrated a UV signature (21/25; 84%) when available. TMB was typical for cutaneous melanoma (median = 9.6 mut/Mb, range 0–35.7). The most frequent other genes with known or likely pathogenic alterations identified included *TERTp* (63%), *CDKN2A* (46%), *TP53* (11%), *PTEN* (8%), *APC* (8%), *ERBB4* (8%), *MITF* (8%), *CTNNB1* (5%), and *MYC* (5%) (Fig. 2b, Supplemental Table 4).

Comparison of tumors sequenced from the primary sites, regional metastases, and distant metastases was performed. Tumors sequenced from primary and regional sites (*n* = 13) versus distant metastases (*n* = 24) showed similar percentages of genomic alterations, including in *TERTp*, *CDKN2A*, *TP53*, and *PTEN* (54% vs. 68%, 39% vs. 50%, 15% vs. 8%, and 15% vs. 4%, respectively).

We identified 327 melanomas (4.6% of the entire melanoma cohort) harboring a missense single nucleotide variant (SNV) mutation in *MAP2K1*. Of these cases, 221 (3.1% of the melanoma cohort) contained a known or likely pathogenic missense SNV, including E203K (*n* = 58), P124L (*n* = 56), P124S (*n* = 45), D67N (*n* = 12), C121S (*n* = 10), F53L (*n* = 8), Q56P (*n* = 6), F53Y (*n* = 6), and K57E (*n* = 4) (Fig. 2a). In contrast to cases with in-frame deletions (Table 2), melanomas with pathogenic missense SNVs in *MAP2K1* contained frequent co-mutation in *BRAF* (58%), *NF1* (23%), and *NRAS* (18%), as well as *TERTp* (80%), *CDKN2A* (60%), and *TP53* (23%) (Supplemental Fig. 1). Median age was 62 years, 67% were male, and most demonstrated a UV signature (165/198; 83%) when available. TMB was elevated (median = 25.2 mut/Mb). Notably, only 16% were triple wild-type (35/221) (Supplemental Fig. 1). These cases featured occasional additional pathogenic genomic alterations in genes in RAS signaling activation, including *ARAF* missense mutations (*n* = 2), activating *RAF1* fusion (*n* = 2), *MAPK1* amplification (*n* = 1), and *KIT* amplification (*n* = 1). Three of the four cases with two pathogenic *MAP2K1* alterations were triple wild-type; the remaining case harbored a *BRAF* L597R mutation.

**Table 2:** Comparative demographics, genomic alterations, and additional biomarkers of melanomas stratified by *MAP2K1* mutation status, with *p* values.

	*MAP2K1* in-frame del	*MAP2K1* missense mutation	*p* value (in-frame del vs missense)	*MAP2K1*-wt melanoma	p value (in-frame del vs wt)
Number of cases	37	221		6851	
% male	49%	67%	0.0385	61%	0.24
Age (range)	66 (28–89)	62 (4–89+)	0.62	63 (<1–89+)	0.59
% Triple wild-type	100%	16%	**<0.0001**	26%	**<0.0001**
% UV signature	84%	83%	1.0	85%	1.0
Median TMB (Q1-Q3; mut/Mb)	9.6 (6.1–17.4)	25.2 (13.1–46.3)	**<0.00001, Mann**–**Whitney** ***U*** **test**	11.3 (3.8–29.6)	0.53
*BRAF* GA	0%	58%	**<0.0001**	37%	**<0.0001**
*BRAF* V600E GA	0%	19%	**0.0013**	22%	**0.0002**
*BRAF* non-V600E	0%	39%	**<0.0001**	16%	**0.0026**
*NRAS* GA	0%	18%	**0.0023**	25%	**<0.0001**
*NF1* GA	0%	23%	**0.0002**	19%	**0.001**
*TERTp* GA	63%	80%	0.02	55%	0.40
*CDKN2A* GA	46%	60%	0.11	41%	0.62
*TP53* GA	11%	23%	0.13	22%	0.11
*PTEN* GA	8%	15%	0.44	13%	0.62
*APC* GA	8%	4%	0.39	4%	0.19
*CTNNB1* GA	5%	5%	1.0	5%	1.0
*ERBB4* GA	8%	7%	1.0	4%	0.19
%MSI	0%	0%	1.0	<0.1%	1.0

The Bonferroni correction for multiple simultaneous comparisons was applied; rows with a significant *p* value (<0.003) are in bold.

*GA* genomic alterations, *del* deletion, *Q* quartile.

Other genomic alterations of *MAP2K1* were rare in the internal series of melanomas. Seven cases with various in-frame *MAP2K1* indels were identified: Q45_F53>L, Q46_T55>H, Q46_K59>L, Q46_E62>L, F53_Q58>L, Q56_G61>R, and L98_K104>M. All cases were of known cutaneous primary. One case had a concurrent *BRAF* L597R mutation, and one case had two truncating *NF1* mutations (*NF1* R440* and *NF1* W1662*).

Three cases had *MAP2K1* amplification, each with copy number ranging from 8 to 10. One case was sequenced from primary cutaneous site, one from colon metastasis of cutaneous primary, and another from liver metastasis of unknown primary site. One case had a concurrent *BRAF* V600E mutation, another had *NRAS* amplification, and the last had two truncating *NF1* mutations (*NF1* L1480fs*2 and *NF1* Y1783*). No pathogenic large structural rearrangements of *MAP2K1* were identified in any case.

Four cases harbored a truncating mutation in *MAP2K1* (Q10*, Q153*, V173fs*2, and Y300*) and three cases had splice site variants in *MAP2K1* (Splice Site 1023–1G>A, Splice Site 1068+1G>A, and Splice Site 961–1G>A). Each of these variants of uncertain significance occurred in *BRAF*, *NRAS*, or *NF1* mutant melanomas.

ctDNA was evaluated from blood specimens (“liquid biopsies”) in a cohort of 307 melanoma patients. Genomic alterations in *MAP2K1* were identified in five patients (Supplemental Table 2). A single case of stage IV melanoma of unknown primary was identified to have an in-frame deletion in *MAP2K1* (E102_103del) at allele frequency (AF) of 3.85%.

## Discussion

While previously reported SNVs of *MAP2K1* in melanoma have been characterized as secondary oncogenic drivers typically paired with common driver mutations of the MAPK pathway, *MAP2K1* deletions, as described here, delineate a unique subset of triple wild-type melanoma (2.0% of all triple wild-type melanoma in this cohort). We propose that the lack of co-mutations in MAPK-activating oncogenic drivers, including those involving *RAF*, *RAS*, and *NF1*, and prior studies characterizing particular functional subtypes of *MAP2K1* mutation, support *MAP2K1* in-frame deletions as defining for a distinct subgroup of melanomas, with potential therapeutic implications.

Several in vitro and in vivo studies have demonstrated that *MAP2K1* in-frame deletions, identical to those in this study, promote activation of the MAP kinase pathway [15, 16, 26–28] (Supplemental Table 3). In particular, recent mechanistic studies by Gao et al. have characterized three distinct classes of mutations in *MAP2K1* (*MEK1*): Class 1 mutants depend on phosphorylation by RAF enzyme for activity, Class 2 mutants show continuous activity independent from RAF stimulation but may be further activated by RAF, and Class 3 mutants demonstrate activity in an entirely RAF-independent manner [16]. Class 1 (RAF dependent) *MAP2K1* mutations consist of various point mutations, Class 2 (RAF regulated) mutations consist mostly of point mutations in various positions as well as deletions spanning the 51–58 positions encoding the negative regulatory region, and Class 3 (RAF independent) mutations consist of deletions involving the 98–104 positions encoding the kinase domain [16]. While the majority of Class 1 (RAF dependent) *MAP2K1* mutations are associated with co-mutation in *BRAF*, *NRAS*, or *NF1*, only a small proportion of class 2 (RAF regulated) cases and no class 3 (RAF regulated) cases share these co-mutations in a limited pan-cancer analysis [16].

Most of the melanomas with *MAP2K1* in-frame deletion in our study, with the exception of 6 cases with Q58_E62del, involve the kinase domain locus corresponding to Class 3 (RAF independent) *MAP2K1* mutations. Analogous to the reports from Gao et al., none of our cases with deletions had co-mutation involving RAF, RAS, or NF1 proteins [16]. This close correlation with prior functional studies and the independence from other MAPK driver mutations support the concept that *MAP2K1* in-frame deletions characterize a distinct subtype of triple wild-type melanoma.

Previously reported genomic alterations in *MAP2K1* in melanoma have consisted almost entirely of point mutations, with only a single report of in-frame deletion [12], and generally have been characterized as secondary driver mutations. Hodis et al. reported that, out of 121 melanomas, seven cases had *MAP2K1* mutations, composed of point mutations and a single in-frame deletion; while four co-occurred with *BRAF* mutation, one co-occurred with *NRAS* mutation, and two occurred independently of *BRAF*, *NRAS*, and *NF1* mutation, co-mutations were not specified by *MAP2K1* mutation type [12]. In a genome-wide sequencing study of 183 melanomas, Hayward et al. reported five cases with *MAP2K1* mutations, all defined as missense in supplemental materials, and all co-occurring with either *BRAF*, *NRAS*, or *NF1* mutations [4]. In another study of 127 melanomas, eight had *MAP2K1* point mutations and two had *MAP2K2* point mutations [10]; these alterations were associated with constitutive ERK phosphorylation, and most co-occurred with either *BRAF* or *NRAS* mutation. Other series revealed *MAP2K1* point mutations in two of 253 melanomas [29] and *MAP2K1* mutations of unspecified type in 21 of 356 melanomas [30]. A case report of cutaneous melanoma metastases sampled over time revealed a *MAP2K1* (F53Y) missense mutation paired with a *BRAF* fusion [11]. The authors of this study reviewed the TCGA database and identified *MAP2K1* alterations in 7% of melanomas, consisting mostly of point mutations with some amplifications and no in-frame deletions, with nearly all co-occurring with *BRAF* or *NRAS* mutations [11]. These various point mutations in *MAP2K1* have largely corresponded to alterations described above as Class 1 (RAF dependent) or Class 2 (RAF regulated) [16].

With regard to BRAF inhibitor resistances, Gao et al. argue, based on their findings and prior study [31], that Class 1 *MAP2K1* mutations are unlikely to contribute to RAF inhibitor resistance, while Class 2 *MAP2K1* mutations, which have been associated with acquired BRAF inhibitor resistance [32, 33], promote RAF inhibitor resistance through RAF-independent enzymatic activity [16]. Class 3 mutations, independent of *BRAF*, *RAS*, and *NF1* mutations, promote the permanently activated confirmation of the MEK1 enzyme. To our knowledge, there is limited clinical data regarding RAF inhibition of Class 3 mutants given the lack of associated *BRAF* mutation, the essential indication for therapeutic RAF inhibition.

Some experimental models have investigated MEK inhibitors for *MAP2K1* in-frame deletions [15, 16, 26, 27]. Gao et al. reported in vitro experiments where Classes 1 and 2 *MAP2K1* mutants were effectively inhibited by allosteric MEK1 inhibitors, while Class 3 mutants, represented by in-frame deletions, were resistant [16]. They further showed that a new experimental MEK inhibitor, which competes for ATP binding to MEK protein, was effective against all three classes. Others models have demonstrated sensitivity of cancer cells with *MAP2K1* in-frame deletion to MEK inhibition [15, 26–28]. Perhaps analogous to in-frame deleted cases, other investigations have shown *MAP2K1* point mutations associated with RAF-independent MEK activity (involving helix-A) promote resistance to both BRAF and MEK inhibition, indicating a need for alternative therapies, such as ERK inhibitors [34].

While prior in vitro and clinical studies have correlated *MAP2K1* missense mutations with resistance to targeted inhibitors in the context of *BRAF*-mutated melanomas [10, 32, 35–41], targeted therapy for melanoma with *MAP2K1* in-frame deletion has not been reported to our knowledge. Case reports of MEK inhibitory therapy for patient with LCH with *MAP2K1* p.E102_103del alteration showed lasting clinical improvement of lesions [42, 43], while a case of colonic adenocarcinoma with the same deletion showed progression on MEK and ERK inhibitory therapy [44] (Supplemental Table 3). In one report, a patient with triple wild-type (*BRAF*/*NRAS*/*NF1* WT) metastatic melanoma with two missense mutations in *MAP2K1* showed a partial response to MEK inhibitor trametinib, followed by progression of disease after two months [45].

Insight into *MAP2K1*-mutated tumors may inform the therapeutic approach to emerging small molecule inhibitors, such as ERK inhibitors [46, 47]. Future clinical studies are needed to identify effective therapies optimized for the various mutational patterns of *MAP2K1*.

Beyond melanoma, *MAP2K1* deletions have been reported rarely among pigmented epithelioid melanocytoma (PEM), deep penetrating nevi (DPN), and Spitz tumors, while various *MAP2K1* alterations have been identified in a range of non-melanocytic neoplasms. In a study of 13 cases of PEM, Cohen et al. characterized two cases with *MAP2K1* in-frame deletions [48]. Interestingly, these *MAP2K1* altered cases did not show co-occurring *PRKAR1A* mutations or *PRKCA* fusions, despite loss of PRKAR1a expression by immunohistochemistry [48]. Isales et al. also noted a case of PEM with *MAP2K1* in-frame deletion, exclusive from *PRKCA* fusion, *NTRK1*/*NTRK3* fusion, and *BRAF*/*NRAS*/*NF1* mutations seen in the other cases [49]. In a thorough characterization of DPN, six cases showed in-frame deletion of *MAP2K1*, all co-occurring with *CTNNB1* mutation, and all mutually exclusive from *BRAF* and *HRAS* mutations [28].

Interestingly, a recent study characterized a subset of Spitzoid melanocytic tumors with activating structural alterations in *MAPK* genes [50]. In addition to in-frame fusions of *MAP3K8*-*DIPC2, MAP3K8*-*PCDH7*, *MAP3K8*-*UBL3*, *MAP3K8*-*SVIL*, and *ATP2A2*-*MAP3K3*, one case showed an in-frame deletion of *MAP2K1*. This *MAP2K1*-mutated case consisted of an atypical Spitz tumor on the leg of a 30-year-old man with dermal growth, expansile nests, associated epidermal hyperplasia, epithelioid cytomorphology with extensive pigmentation, moderate nuclear atypia, and low mitotic rate. Follow-up showed no recurrence at 9 months [50].

With respect to histomorphology, the melanomas in this series were diverse. The qualities shared by the primary tumors, including exophytic nodules, irregular nested bases, and nuclear pleomorphism, are common to many cutaneous melanomas with tumorigenic vertical growth phases. In addition, the cytomorphology varied, with most displaying epithelioid morphology and a small minority showing spindled and rhabdoid cytomorphology. We note that a small proportion of cases were pigmented, although our analysis did not reveal tendency towards pigmented epithelioid cytomorphology as noted in previous study of *BRAF*-mutated melanomas [51]. Furthermore, we did not observe characteristic PEM-like, DPN-like, or spitzoid cytomorphology. Additional study and accumulation of increased numbers of primary cases may allow for improved recognition of common morphologic features.

*MAP2K1* mutations occur in ~20% of cases of LCH and are mutually exclusive from *BRAF* mutation, the most common pathogenic mutation in LCH [13–15, 52]. These *MAP2K1* mutations in LCH are predominantly in-frame deletions (corresponding to either class 2 or class 3 *MAP2K1* mutations), with a minor component of point mutations [13–15, 53]. McGinnis et al. reported that, of their five cases of LCH with *MAP2K1* mutation, two also had *BRAF* V600E mutation [54]. Interestingly, these authors describe that these two *BRAF*/*MAP2K1* co-mutated cases had point mutations in *MAP2K1*, whereas the *MAP2K1* in-frame deletions were apparently exclusive from *BRAF* mutants [54].

A similar mutual exclusivity of *BRAF* and *MAP2K1* mutations has also been reported in other neoplasms, including hairy cell leukemia (HCL) and various epithelial malignancies. While classical HCL harbors *BRAF* V600E in nearly all cases, ~30% of variant HCL has *MAP2K1* mutation [55–57]. These *MAP2K1* mutations in HCL consist mostly of point mutations, with rarely reported in-frame deletion [56, 57]. *KRAS* and *MAP2K1* point mutations have been shown to occur in a mutually exclusive manner in Rosai–Dorfman disease [58]. *MAP2K1* mutation has been identified among subsets of smoking-associated lung carcinomas (mutually exclusive from *EGFR*, *BRAF*, *KRAS*, and *NRAS* mutations) [59, 60], lung adenocarcinoma in situ and early invasive disease [61], and *KRAS/NRAS/BRAF/PIK3CA* wild-type (“quadruple-wild-type”) colorectal carcinomas [62]. Study of extracranial arteriovenous malformations revealed point mutations and, rarely, in-frame deletions of *MAP2K1* [63].

Our study also provides a proof of concept that liquid biopsy can detect ctDNA of in-frame deletion of *MAP2K1* in melanoma, with a single case showing this alteration. Four additional melanoma cases demonstrated missense mutations in *MAP2K1*. Liquid biopsy may be a valuable method in these tumors, and further investigation may be warranted.

With respect to limitations, the cases described here were collected from patients with advanced malignancies, submitted for detection of therapeutically targetable mutations. Thus, these cases likely exemplify the aggressive end of the biologic spectrum without representing early, thin, and/or indolent melanomas. We speculate that we did not identify PEM-like, DPN-like, or atypical Spitz tumors as previously reported [49, 50], because these tumors are typically not aggressive and are unlikely to be submitted for therapy-oriented genomic sequencing. Another limitation of this study is that, owing to the nature of our sample bank, we were unable to directly assess MAPK activation in our particular cases. Nevertheless, three lines of evidence support our inference that the in-frame deletions of *MAP2K1* in our melanoma cases were activating. First is extensive mechanistic literature that *MAP2K1* in-frame deletions identical to those in our study activate MAPK signaling [15, 16, 26–28] (Supplemental Table 3). Second is evidence of MAPK pathway activation in prior study of non-melanoma tumors with these same *MAP2K1* in-frame deletions [15] (Supplemental Table 3). The third line of evidence is the mutual exclusivity that we found in our cohort from well-characterized mutations of the MAPK pathway (Fig. 2b, Table 2). Follow-up data, which was not available in this study, will be important to obtain for this cohort of *MAP2K1*-mutated melanoma to correlate with prognosis and therapeutic outcomes. In particular, therapeutic responses of this subgroup to inhibitors of the MAP Kinase pathway, including MAP2K1 and ERK inhibitors, require further study. With respect to *MAP2K1* missense mutations, it is possible that the *BRAF*-mutated cases, through the effects of combined BRAF and MEK inhibitor therapy, are enriched for these point mutations of *MAP2K1*, selected through acquired resistance. Finally, while H&E slides and accompanying pathology reports were reviewed from all cases, we were not able to review immunohistochemical slides, limiting our ability to correlate genomic findings with immunophenotype.

Melanomas with *MAP2K1* in-frame deletions show distinct mutational profiles and correlate closely with prior functional studies of *MAP2K1* deletion. Additional studies on therapeutic approaches to *MAP2K1*-mutated melanomas are needed.

## Supplementary information

Supplemental Figure 1

Supplemental Table 1

Supplemental Table 2

Supplemental Table 3

Supplemental Table 4
