# Supplementary material for: Melanoma with in-frame deletion of MAP2K1: a distinct molecular subtype of cutaneous melanoma mutually exclusive from BRAF, NRAS, and NF1 mutations
Source: Mod Pathol. 2020 Jun 1;33(12):2397–406. doi: 10.1038/s41379-020-0581-5 (PMC7685971; doi:10.1038/s41379-020-0581-5)
Supplement: Supplementary file 1 — Supplemental Figure 1 [file 41379_2020_581_MOESM1_ESM.pdf]

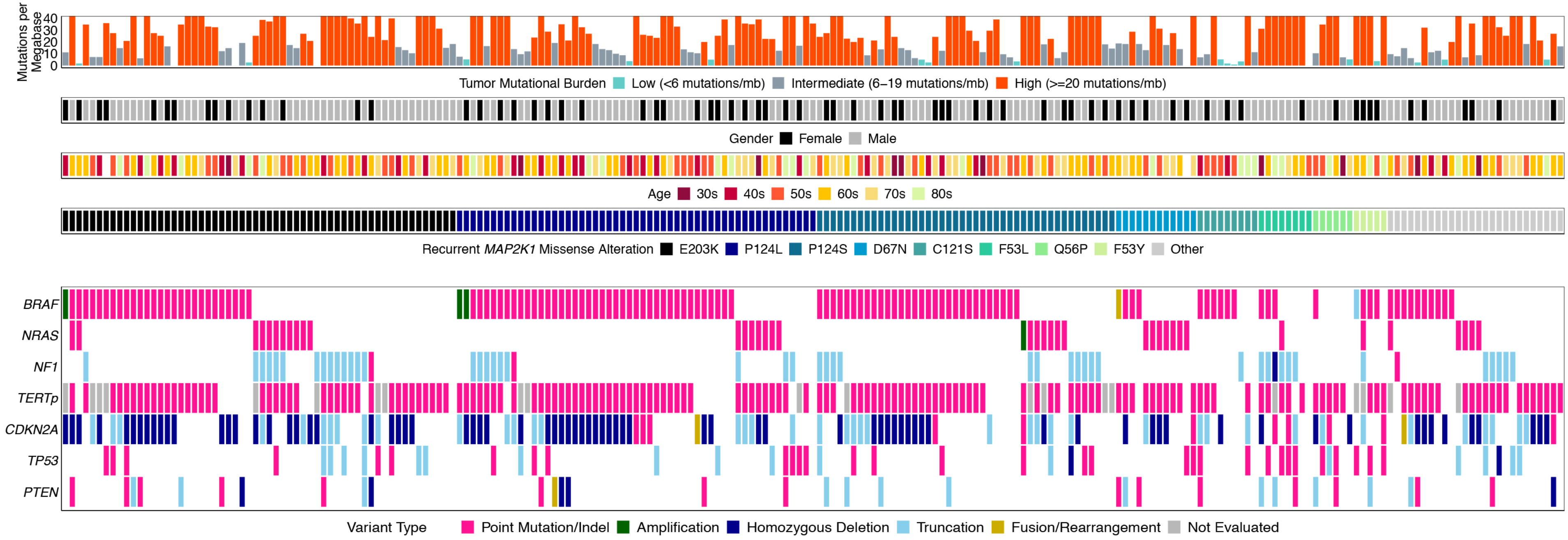

Supplemental Figure 1. Summary of clinical features and molecular alterations in melanomas with known and likely pathogenic *MAP2K1* missense mutations.
