# Supplementary material for: Melanoma with in-frame deletion of MAP2K1: a distinct molecular subtype of cutaneous melanoma mutually exclusive from BRAF, NRAS, and NF1 mutations
Source: Mod Pathol. 2020 Jun 1;33(12):2397–406. doi: 10.1038/s41379-020-0581-5 (PMC7685971; doi:10.1038/s41379-020-0581-5)
Supplement: Supplementary file 4 — Supplemental Table 3 [file 41379_2020_581_MOESM4_ESM.pdf]

Supplemental Table 3. *MAP2K1 (MEK1)* in-frame deletions with respective number of melanoma cases identified, and corresponding allele-specific literature review to include functional study data, genomic profile of previously reported melanocytic neoplasm cases, and clinical response to targeted therapeutics in available tumor types.

*Pan-cancer studies that do not specify tumor types are not included.

Abbreviations: LCH, Langerhans cell histiocytosis; DPN, deep penetrating nevus; del, in-frame deletion; PEM, pigmented epithelioid melanocytoma.

***MAP2K1* Mutation**	**# cases**	**Studies with functional characterization**	**Genomic profile of previously published melanocytic neoplasms***	**Pan-cancer known treatment and follow-up data**
**E102_I103del**	11	❖ Gao 2018:<br>▪ Multiple *in vitro* models expressing this *MAP2K1* del show constitutive MAPK activity, which is RAF and phosphorylation independent and insensitive to feedback.<br>▪ High levels of ERK output and transformation in untransformed cells.<br>▪ Unresponsive to current MEK inhibitors, which bind the inactive conformation of the enzyme; ATP-competitive MEK inhibitor suppresses activity.<br>❖ Yuan 2018:<br>▪ Fibroblasts and 293T cells with this *MAP2K1* del show enhanced MEK homodimerization, promoting intradimer cross-phosphorylation of the activation loop.<br>▪ Conferred variable resistance to MEK inhibitors both *in vitro* (293T and fibroblasts with MEK1 mutant) and *in vivo* (MEK1 mutant melanoma xenograft) with sensitivity to trametinib noted for E102_I103del.<br>❖ Chakraborty 2014:<br>▪ HEK293 cells transfected with this *MAP2K1* del show constitutive ERK1/2 phosphorylation.<br>▪ LCH cells show MEK1 and ERK1/2 phosphorylation by imaging flow cytometry.<br>▪ MEK inhibitor (U0126) but not vemurafenib inhibited ERK phosphorylation in *MAP2K1*-indel HEK293 cells and LCH cells.<br>❖ Kohsaka 2020:<br>▪ Increase ERK phosphorylation in 293T cells expressing this *MAP2K1* del.<br>▪ Ba/F3 cells, an IL-3 dependent cell line, grow without IL-3. MEK inhibitors inhibit growth *in vitro* and in 3T3 cells in nude mice.<br>❖ Yeh 2017: 293FT cells with this *MAP2K1* del show elevated ERK phosphorylation that was inhibited by MEK inhibition.	❖ Yeh 2017: 1 case of DPN wildtype for *BRAF*, *NRAS*, *HRAS* and other *MAP2K1* alterations<br>❖ Hodis 2012: 1 case of melanoma, co-mutations not distinguished between dels and point mutations of *MAP2K1*	❖ 46 yo M with LCH: remission with MEK inhibition (trametinib) (Papapanagiotou 2017);<br>❖ 18 yo M with LCH: response to MEK inhibition (trametinib) (Lorillon 2018);<br>❖ 52 yo F with colon adenocarcinoma: response limited to decreased serologic markers with subsequent progression on MEK (trametinib) and ERK (ulixertinib) inhibition (Wang 2019)
**P105_A106del**	8	❖ Yuan 2018: results similar to E102_I103del, described above<br>❖ Kohsaka 2020:<br>▪ Increased ERK phosphorylation in 293T cells with this *MAP2K1* del<br>▪ Ba/F3 cells, an IL-3 dependent cell line, grow without IL-3. MEK inhibitors inhibit growth *in vitro*		
**Q58_E62del**	6	❖ Chakraborty 2014: results similar to E102_I103del, described above<br>❖ Yeh 2017: results similar to E102_I103del, described above	❖ Cohen 2017: 2 cases of PEM, mutually exclusive from *BRAF* mutation<br>❖ Yeh 2017: 1 case of DPN wildtype for *BRAF*, *NRAS*, *HRAS* and other *MAP2K1* mutations	
**I103_K104del**	5	❖ Gao 2018: results similar to E102_I103del, described above<br>❖ Yuan 2018: results similar to E102_I103del, described above<br>❖ Yeh 2017: results similar to E102_I103del, described above	❖ Isales 2019: 1 case of PEM, exclusive from *BRAF*, *NRAS*, *NF1* mutation<br>❖ Quan 2019: 1 Spitzoid neoplasm with no other identified alterations<br>❖ Yeh 2017: 2 cases of DPN wildtype for *BRAF*, *NRAS*, *HRAS* and other *MAP2K1* mutations	
**I99_K104del**	3	❖ Gao 2018: results similar to E102_I103del, described above		
**L98_I103del**	3	❖ Gao 2018: results similar to E102_I103del, described above		
**E41_F53del**	1			
